# Patents of Interest to Dentists, Recently Granted

**Published:** 1906-05

**Authors:** 


### PATENTS OF INTEREST TO DENTISTS, RECENTLY GRANTED.

815907, Dental expander or dilator Cleveland G. Davis, Manistee, Mich.
815807, Dental plate swage, Grant E. Freeborn, Belfast, N. Y.
815534, Toothpick and forming same, Henry S. Hopper, Norfolk, Va.
815374, Tooth mold, Evelyn Pierrepont, London, England.
816069, Dental tool holder or clutch, Benoni S. Brown, Onset, Mass.

816481, Cap and brush for bottles, Johnson Lane, Jamestown, Cal.

817050, Dental floss holder, Joseph C. De La Cour, Conshohohocken, Pa.

12470, Manufacture of acid-proof cement, Richard Liebold, Weimar, Germany.

816668, Dental instrument for raising barbs upon wires, Lloyd S. Lourie, Chicago, Ill.

816685, Shield and moistener for dental tools, Charles A. Sevier, Jackson, Tenn.

816828, Dental contour pliers, Nathan H. Smith, Seattle, Wash.

817978, Toothpick, Albert E. Lickman, Baltimore, Md.

818000, Tooth brush, Charles R. Stevenson, Boston, Mass.

Copies of above patients may be obtained for ten cents each, by addressing John A. Saul, Solicitor of Patents, Fendall, Building Washington, D. C.

